# Barriers and facilitators to adherence to anti-diabetic medications: Ethiopian patients’ perspectives

**DOI:** 10.4102/phcfm.v9i1.1411

**Published:** 2017-10-17

**Authors:** Bruck M. Habte, Tedla Kebede, Teferi G. Fenta, Heather Boon

**Affiliations:** 1 School of Pharmacy, College of Health Sciences, Addis Ababa University, Ethiopia; 2 School of Medicine, College of Health Sciences, Addis Ababa University, Ethiopia; 3 Lesley Dan Faculty of Pharmacy, University of Toronto, Canada

## Abstract

**Background:**

Little is known about the experiences of Ethiopian patients with type 2 diabetes related to adherence to their anti-diabetic medications. This may limit attempts to develop and implement patient-centred approaches that consider Ethiopian contexts.

**Objectives:**

To conduct an exploratory study with a focus on identifying barriers and facilitators to anti-diabetic medications adherence in Ethiopian patients with type 2 diabetes.

**Methods:**

Qualitative methods were used to conduct semi-structured interviews with 39 purposively selected participants attending clinic in three public hospitals in central Ethiopia. Open coding was used to analyse the data to identify key themes.

**Results:**

A number of factors were identified as barriers and facilitators to participants’ adherence to their anti-diabetic medications. The most common factors were perceptions related to their illness including symptoms, consequences and curability; perceptions of medications including safety concerns, convenience and their necessity; religious healing practices and beliefs; perceptions about and experiences with their healthcare providers and the healthcare system including the availability of medications and diabetes education; and finally perceived self-efficacy and social support.

**Conclusions:**

The findings of this study provide guidance to strengthen diabetes education programmes so that they reflect local patient contexts focusing among other things on the illness itself and the anti-diabetic medications.

## Introduction

Diabetes mellitus, described as ‘one of the largest global health emergencies of the 21st century’, is among the non-communicable diseases that continue to rapidly increase in numbers and significance especially in the developing world. The African region is home to around 14.2 million people with diabetes of which 1.3 million are in Ethiopia. These figures are expected to rapidly increase along with increases in urbanisation and life expectancy.^1^ Type 2, which is the most common type of diabetes, has been associated with rapid economic development, increasing urbanisation, ageing populations, reduced physical activity and unhealthy diets. Optimal self-management with non-pharmacological and pharmacological therapy along with appropriate healthcare provider support enables people with diabetes to live a long and healthy life. On the other hand, poorly managed diabetes leads to serious complications and a shortened life.^1^ The positive impact of anti-diabetic medications in the blood glucose control of type 2 diabetes and the associated reduction of microvascular and macrovascular complications have long been established.^2,3,4^

Nevertheless, adherence to recommended medication regimens is a challenge with poor adherence associated with increased blood glucose levels, suboptimal diabetes outcomes including diabetes related complications, more hospital admissions and increased medical care costs.^5,6,7,8,9,10^ Common factors that have been cited for the suboptimal adherence levels among Ethiopians include consulting traditional healers and being on oral anti-diabetic medications^11^; concerns about potential and experienced side effects and lack of financial resources^12,13,14^; complexity of medication regimens; and experience of depressive symptoms and ‘medium’ level of diabetic knowledge.^15,16^ These studies, however, all utilised quantitative methods and were unable to provide in depth insight into patients’ perceptions and experiences of adherence to their recommended medication regimens. They may therefore be of limited use in informing patient-centred approaches that could be used to improve adherence. The aim of this study was to conduct an exploratory study using qualitative methods to elicit the barriers and facilitators to adherence to recommended anti-diabetic regimens of patients with type 2 diabetes.

## Methods

### Design

A qualitative research design with in-depth interviews was used for this study.

### Study setting

Study sites were three public hospitals in central Ethiopia. Two of these, Tikur Anbessa Specialized Hospital (hereafter referred to as Tikur Anbessa) and Yekatit 12 Hospital (hereafter referred to as Yekatit 12), are located in Addis Ababa (the capital and largest city of Ethiopia). The third study site was Butajira Hospital (hereafter referred to as Butajira), the only public hospital in Butajira, which was included to explore the perceptions of patients living in the peri-urban part of the country. A brief description of Addis Ababa and Butajira is given in Table 1.

**TABLE 1:** Description of study settings.

Variables	Addis Ababa	Butajira
Significance	Largest urban centre in the country	Home to Addis Ababa University’s demographic surveillance site
Population^17^	3.2 million	63 000 000
Ethnic groups^18^	Amhara (47%), Guragie (16.3%), Oromo (19.5%) and Tigrie (6.2%)	Guragie (82%)
Religions^18^	Orthodox Christianity (74.7%), Islam (16.2%) and Protestant Christianity (7.8%)	Islam (51.3%), Orthodox Christianity (39.6%) and Protestant Christianity (8.7%)
Literacy^18^	85.3%	37.9%

Tikur Anbessa is a teaching hospital which is also the highest referral hospital in Ethiopia. Patients who are diagnosed with diabetes are seen in the diabetes centre that was run by three endocrinologists and two endocrinology fellows who work as consultants on a rotating basis during the study period, around six internal medicine residents assigned to take the primary role in managing the patients during their month-long attachments, six nurses and one recently recruited pharmacist. Yekatit 12 is a general hospital that is managed by the city administration and has recently started training medical doctors. The clinical services for patients with diabetes were primarily given in the general outpatient department that was run at the time by four general practitioners. Cases that were considered to be in need of specialist care were referred to the medical clinic run by internists on a rotating basis. Patients attending treatment in Tikur Anbessa and Yekatit 12 were ‘randomly’ assigned to an attending doctor each time they came for their appointment. Butajira is a general hospital that managed the treatment of patients in a similar pattern as those of the other two hospitals. However, it has lately established a separate medical clinic run by a general practitioner and a nurse that serves patients with diabetes. During the time of the study, these patients had just started to meet the same doctor when they came for their monthly appointment.

### Study participant recruitment

Participants were purposively selected patients with type 2 diabetes who were attending their treatment for the duration of the study in the selected hospitals. The inclusion criteria were as follows: patients must be aged 18 years or older, be on anti-diabetic medications for at least 1 year and have no known or overt psychiatric problems. The only exclusion criterion was being a healthcare professional. Apart from these, efforts were made to purposively select patients with a wide variation in terms of socio-demographic characteristics (such as sex, age, marital status, educational level, religious affiliation, employment status and place of residence), income level and illness duration.

### Interview methods

Individual in-depth interviews that had a median duration of 49 min (and ranged from 30 to 120 min), were conducted from December 2013 to March 2014 by the first author. The interviews (completed in Amharic) were audio recorded with the participants’ consent. The interview questions focused on the anti-diabetic medication-taking experiences of study participants with special emphasis on the barriers and facilitators to participants’ adherence to recommended regimens. The interview guide that was originally prepared in English was translated to Amharic and back to English to check its consistency before the Amharic version was used.

### Data analysis

The interview data were transcribed in Amharic into Microsoft Word by an experienced research assistant. The first author checked the quality of interview transcripts by listening to randomly selected audios from each study site while reading the transcripts. Interview transcripts were then read repeatedly before using open coding to identify key concepts and classify them into separate categories and sub-categories. Data collection and analysis continued in an iterative process until all key themes were saturated and no new information was emerging from the interviews.^19^ Initial coding and categorisation was done in Amharic which was followed by further analysis and interpretation that was carried out after translating key components of the interview transcripts that were relevant to the emerging themes into English. In this regard, the first author and the last author worked together to analyse and interpret key findings until they reached consensus. NVivo 10 qualitative data analysis software was used to manage the data.

### Ethical considerations

This study received ethics approval from the Institutional Review Board of the College of Health Sciences, Addis Ababa University (protocol number 036/13/PSP). All the respective health institutions also gave permission for the data collection. Furthermore, all the participants who took part in the study gave their informed consent while their anonymity was maintained.

## Results

### Demographics of study participants

In total, 45 individuals were invited to participate in this study of whom 39 participated. The six who were not able to participate cited personal reasons or there were problems in gaining access to them via telephone. Of the study participants, 24 were from Addis Ababa attending treatment at Tikur Anbessa and Yekatit 12 (12 from each site), and the remaining were from Butajira town or its environs. Most of the study participants were on either a combination of glibenclamide (popularly referred to using the innovator brand name of Daonil) and metformin (22/39) or insulin (14/39). Close to two-thirds of participants were enrolled in a government care programme that allowed free medical treatment and medications for those who were determined to be of ‘very low’ economic status. Thirteen of the participants reported having to pay out of pocket for their treatment including medications. Table 2 summarises relevant demographic and treatment characteristics of the study participants. Notable differences were not evident in the perceptions and experiences of the participants from the different sites, and thus the findings are presented as a single set.

**TABLE 2:** Study participants’ characteristics (*n* = 39).

Characteristics	Number
**Sex**	
Female	19
Male	20
**Age (years)**	
30–39	2
40–49	8
50–59	14
60–69	10
> 70	5
**Educational status**	
Low education status (illiterate or basic literacy)	16
Elementary complete	8
Secondary school complete	8
Post-secondary school education	7
**Diabetes duration (years)**	
1–5	10
6–10	14
11–15	7
16–20	4
21–25	4
**Payment for health services including medicines**	
Out of pocket	13
Government	23
Employer	3
**Treatment regimen**	
Oral:	
Glibenclamide + metformin	22
Glimepiride + metformin	1
Glibenclamide	1
Insulin	14
Insulin + metformin	1

### Factors affecting recommended medication-taking

Although the majority of the participants claimed to be adherent to the recommended medication regimen, almost all described at least some times when they were non-adherent and a few openly expressed their non-adherence, explaining that they take their medication only when ‘required’.

A number of barriers and facilitators related to adherence were cited by the study participants. The most common factors included illness perceptions, especially related to symptoms, consequences and the curability or controllability of diabetes; medication-related perceptions, including the necessity of taking medication, concerns about adverse effects and convenience; religious healing practices and beliefs; perception about healthcare providers; healthcare system-related perceptions such as the availability of medications and presence of diabetes education; and finally self-efficacy of patients and social support from family and others close to participants. It was apparent that usually multiple factors impacted medication adherence for each participant.

#### Illness perceptions

Illness perceptions were a key factor that seemed to influence participants’ decisions to adhere to recommended medications. Most notable among these were patients’ perceptions regarding the severe consequences and incurability of diabetes. Some cited experiences with close family members who have lived and died with diabetes or experienced complications as shaping their perceptions.

‘Diabetes leads to many different complications if one does not take precautions - it could paralyse and shut off the brain. … The medicine would never be discontinued … I take the smaller medicine 30 minutes before eating my meal, I eat my meal and take the bigger one. It wouldn’t be discontinued even if there is fasting.’ (Female, elementary school complete, 9 years with diabetes)

The appearance of severe diabetes related symptoms led some participants who had abandoned treatment early on to restart it.

‘Had I been able to follow [*my regimen*] strictly, I think it [*my illness*] could have been in a better position even if it would not go away. But I abandoned it for 8 months [*in favour of holy water*] and my body became so horrible and myself so ugly looking. I literally became a cloth on a wooden pole. I gave all my clothes to my relatives and I am now buying all over again now that my body has regained some of its old self.’ (Female, high school graduate, 12 years with diabetes)

There were some participants who delayed initiation of their treatment for diabetes largely because of a lack of acceptance of their diagnosis. This may have been because of their being asymptomatic, having limited knowledge about diabetes or the fact that diabetes was sometimes diagnosed by chance while seeking treatment for another condition.

‘At the beginning I had hypertension and then I went to have treatment for my haemorrhoid. That was when they informed me that the tests showed diabetes – a diagnosis I had difficulty to accept. I thought I would die immediately. The doctors present at the time appeased me and urged me to start on medications. But I didn’t start until after 2 years later.’ (Female, diploma holder, 9 years with diabetes)

Illness perceptions, especially related to diabetes symptoms, hope for a cure, and perceived minor consequences were among reasons cited by some study participants for not adhering to their recommended treatment regimen. Non-adherence included abandoning treatment in the early period as well as discontinuing it from time to time, as can be discerned in the following quote.

‘When I was tested, it had reached 400 (mg/dl). They then gave me these medicines. So when it goes down, I would discontinue it, and when it goes up I would restart on the medicine. When I noticed that the increase in the urine frequency and also in the hunger feelings, I would start on the follow up. My follow up fluctuates, it goes up and down and this is how I have been. Today it is 322, last month it was 240 but there is no symptom that I feel. I am taking Daonil.’ (Male, high school graduate, 3 years with diabetes)

#### Medication-related perceptions

**Necessity of treatment:** Some study participants stated their strong commitment to take their medications as per the recommended regimens citing necessity beliefs such as health benefits and the efficacy of their medications. The necessity beliefs were so strong that these participants claimed not to discontinue their medications even if they have to buy it from private sources when they are unavailable at the hospital pharmacy.

‘It [*diabetes*] is still tormenting me. My efforts to get free medical aid from the government were not successful. I am surviving by purchasing [*medicines*] with whatever means I have. After all it is [*my*] life…. It will never be discontinued. I will take even if it means troubling my son for money to buy.’ (Female, low education, 18 years with diabetes)

In contrast, a few participants did not strictly adhere to recommended regimens because of lack of perceived necessity of the medicine in reducing the blood glucose levels or to effect cure.

‘I started taking metformin later on … but what use does it have for me? There is nothing that goes down and I observe no change whatsoever. So … I didn’t take it [*metformin*] even though I brought it [*from the hospital*], I didn’t give it a second thought.’ (Female, Diploma holder, 9 years with diabetes)

‘It is no use taking medicines after the problem has come. Once you get the nerve problem, there can only be a pain killer but no cure for the disease. You have to take precautions beforehand.’ (Male, diploma holder, 8 years with diabetes)

**Safety concerns about medications:** Some study participants expressed concerns about the safety of their medications especially related to hypoglycaemia and abdominal side effects. Such concerns led them to decrease in the dose or discontinue the medications they were taking. Other participants refused to accept recommendations to initiate insulin citing perceptions about the possible complications and adverse effects and associating it with advanced degree of illness severity.

‘I informed the doctor that it has been 15 days since I discontinued the second one [*metformin*] because it was disagreeable to me. It hurt my stomach.’ (Female, high school graduate, 18 years with diabetes)

‘Now they are telling me to move to the injections. But I am resisting it. … I fear the injections very much; even when I think about it. Injections need great care; if the levels go down there is the possibility of falling down and what I think of. I don’t think I will be able to get up again if I fall. I am hypertensive and such complications may make me paralysed.’ (Female, diploma holder, 9 years with diabetes)

**Convenience:** Convenience (or lack thereof) was another factor that participants cited as a reason to miss their medications. Some participants reported resisting recommendations to initiate insulin citing inconveniencies in handling insulin as compared to the oral agents. On the hand, convenience also seems to contribute to one participant’s decision to inject more than the recommended dose of insulin.

‘And if I have to go to the countryside for mourning I don’t carry them [*the oral anti-diabetic medicines*] and would take them after a couple of days. This is my own fault. I don’t take it because it is inconvenient and I don’t have it with me, but this shouldn’t have been the case. In any case I should take precautions whether it is convenient for me or not [*to take my medicines*] but still it wouldn’t kill me at once.’ (Male, diploma holder, 7 years with diabetes)

‘The tablet you can put in your bag and take it wherever you go. But that one [*insulin*] is difficult [*explaining why she resists doctors’ recommendation to start*].’ (Female, diploma holder, 9 years with diabetes)

‘I take Insulatard Denmark, the milky one. I take 20 in the morning and 15 in the evening. Sometimes if I will be staying late at the office, I take the whole dose in the morning. I take metformin 1000 [*milli*] gram in the evening. I have never encountered any lowering [*of sugar levels*] problems. As I take things easy, I have no pain whatsoever.’ (Male, diploma holder, 8 years with diabetes)

#### Religious healing

Some study participants who were followers of Orthodox Christianity described how they practised their religious healing practices and duties in a manner that does not affect their adherence to recommended treatment regimen. This included attending church services after taking their medicines and food, praying and using holy water while still maintaining the recommended dosage schedule. They also reported fasting from selected foods but not abandoning food altogether contrary to what is practised by the strong adherents. Such practices at the very least were observed to bring about psychological benefits to the study participants.

‘Now I fast by abstaining from meat and butter but otherwise eat other food and water. I have to eat at the specified times, it is mandatory [*for the medication*]. But otherwise I fast like everybody else.’ (Female, low education, 7 years with diabetes)

For other participants, religious healing practices were observed to affect the adherence to recommended medication regimens. Notable was the use of holy water instead of prescribed medicines among the followers of Orthodox Christianity especially those visiting holy water sites and for the refusal to initiate on insulin as it was not amenable to discontinuation ‘from time to time’ for religious reasons as are the oral agents. The other religious practice that affected adherence was fasting which was reported by participants who were Orthodox and Protestant Christians and Muslims. Fasting led to reduction in the dose for some, whereas it led to modification of the time schedule for others. For those who continue taking their insulin without food, it may have led to hypoglycaemic incidents.

‘I use holy water that people bring to me as I [*now*] cannot manage to go to the holy water site. I have discontinued the medicine that time when I went to a holy water site at Shenkora.’ (Female, low education, 18 years with diabetes)

‘I fast. Previously I would unknowingly inject the medicine in the morning and spend the day without eating. I stopped this after having read some paper which says that if one does not eat within 30 minutes of insulin injection it would lead to kidney damage. Sometime I would spend the day without eating and injecting. If it is a mass prayer program I might fast until 3 pm, if it is private I might stay until 6 pm. If I have to inject in the interim I might do so in less amount, say half the dose. If it is evening I only take the 40 only.’ (Female, low education, 18 years with diabetes)

#### Healthcare providers

Healthcare providers, especially the relationship and availability of appropriate providers, were identified both as barriers and facilitators to medication adherence. Healthcare providers, especially doctors, were reported to have played a positive role in supporting the adherence, be it in the maintenance of prescribed regimens or initiation of insulin.

‘They prescribe the medicines for restoring health and I take them; I take them correctly at the specified times. They tell me not to stop taking it no matter what, and not to let the time pass. So I take the medicine at the specified times, I am healthy.’ (Female, low education, 20 years with diabetes)

There were some study participants who expressed resistance to the recommendations by doctors to increase doses of anti-diabetic medications which were related to safety concerns but also apparently lacking trust in them and questioning their expertise.

‘Sometimes I feel sick when I take the medicine for my diabetes. I sometimes wonder if I should decrease the medicine [*dose*] but I am afraid [*of the consequences*]. Because, when I [*my sugar level*] am at 70 (mg/dl), the doctors continue to prescribe the same dosage [*two tablets*] … But I plan to decrease it [*the dose*]. If I am okay I would like to take just one while still controlling it. … But I really would like to reduce it. When it [*sugar level*] is 600 (mg/dl) I still take 2 [*tablets*]. There is no change [*in the doses*] to go up or down. I have my sugar levels checked every 15 days even though I hardly afford it. It [*the level*] is okay, never goes beyond 100.’ (Male, high school graduate, 2 years with diabetes)

#### Healthcare system

The healthcare system, especially the provision of diabetes education and the availability of medications, was cited among the factors influencing adherence to medications.

**Availability of medications:** The availability of medicines at the hospital pharmacy was among key factors mentioned for patient adherence. There are few participants who have not encountered problems with the availability but reported concerns were more pronounced among those who depend on free medications (or programmes that allow them to purchase medications for reduced prices) as they could not afford to purchase their medications from sources outside these free or subsidised programmes. This apparently had implications of decreasing medication doses, going by without the medicines and even having to buy expired medicines from illegal sources.

‘Now I get the medicines for free, previously I had to pay for it. No, the medicines are not always available. The smaller one was not available for 4 months. So during that time it has to be bought. Sometimes there are cases when out of fear that I wouldn’t completely get the medicine I would decrease the dose from 4 to 2. So I make it 1 in the morning and evening. No problem with the bigger because it is available.’ (Male, elementary school complete, 15 years with diabetes)

**Diabetes education:** Patients clearly valued education about diabetes from healthcare providers, but many expressed frustration and concern that they had not received sufficient information.

‘I know nothing about diabetes. Let alone the basics, there was no one who instructed me as to how I should inject insulin when I was started on it. … The pharmacy people didn’t educate me. And when I inquired of them as to how I take it citing that I was a novice, they told me to go and ask my doctor. And when I went with my medicines to the doctor, he told me to go and get educated at the health centre and it was not his concern. So in the middle of it I was not going to take the medicines and that was a problem. Should something happen I was the one to be hurt. … See here [*showing the bruise marks on her thighs*] what happened to me, all because I didn’t know how to use insulin.’ (Female, high school graduate, 12 years with diabetes)

‘Yes, I agree on the necessity of the medicine. If I could get a good doctor, one that gives good counselling like the ones that used to be here that counselled. Now this [*present*] ones, they don’t even have written materials. The previous ones [*who used to be there*] used to write about eye problems, about legs, about teeth; foods to be eaten separately and those to be avoided separately in great detail.’ (Female, low education, 8 years with diabetes)

The diabetes education sessions especially offered by the Ethiopian Diabetes Association was commended by some participants who claim to have gained knowledge about the illness and the treatment that supported their adherence to recommended treatment regimen.

‘After attending educational sessions at the association [*Ethiopian Diabetes Association*] I knew what was beneficial and what was harmful. After that I wouldn’t let 5 minutes pass for taking my medications; if I take my medicine at 8 in the morning, I will take it at 8 in the evening.’ (Female, high school graduate, 25 years with diabetes)

#### Patients’ self-efficacy

There were some participants who appear to be confident in modifying their insulin dose. Although some decrease or increase based on the laboratory results of their blood glucose levels, there are others who may just decrease based on subjective feelings or when planning some activities which they believe may further decrease their glucose levels.

‘One should make his own modifications in addition to that of the doctor. I could decrease the medicine by 10 or 5 [*units*]. When it goes up, one also needs to increase [*the dose of the medicine*]. But myself, I take the prescribed dose [*and do not increase*] and it is only when it lowers that I modify. It is of course important to consider the doctor’s advice. Now when I go to my farm or have to travel it goes down. So I take a lower dose and it doesn’t create that much problem. But if I take as per their [*doctors’*] recommendation I could fall.’ (Male, low education, 8 years with diabetes)

There were a couple of study participants who seem to have lower self-efficacy and instead expressed their strict adherence to healthcare providers’ recommendation.

‘I do as the doctor tells me; I don’t deviate from his words and if I do the harm is on me. If I reduce it [*the dose*] I myself will be harmed, if increase it I will still be harmed. Why would I go to a doctor if I decide by myself? This is it.’ (Female, high school complete, 25 years with diabetes)

#### Social support

Social support from among family members, colleagues at work and neighbours was stated as being beneficial for participants’ adherence to recommended medication regimens. Support was in the form of moral support of adherent behaviour, reminding to take medications, availing medications (in kind or providing funds), covering work to avoid falls because of hypoglycaemia and preventing medication-taking when glucose levels were low.

‘Sometimes when there is a problem at home [*such as mourning*], neighbours check and remind if I have taken [*my medications*] or not. … There are small children [*in the house*] who remind me of my breakfast and my medicines. They are constantly doing that. And so I don’t delay taking the injection. And I am at peace now.’ (Female, low education, 20 years with diabetes)

## Discussion

Current evidence shows that a successful diabetes care model requires the active engagement of patients in managing their condition so as to achieve and maintain optimal health outcomes.^20^ Studies to date, most of which are from western cultures, report patient, treatment and health-system-related factors that serve as barriers and facilitators to the successful management of diabetes. However, the few reports from developing countries including Ethiopia tend to focus solely on the barriers and do not give due attention to factors that may facilitate adherence to recommended regimens. This study is among the few from the Ethiopian setting and to our knowledge the only one to report in depth on both the barriers and facilitators to adherence to anti-diabetic medications. The major factors that were identified in this study included participants’ perceptions about their illness such as symptoms, consequences, curability and treatment using religious healing practices and perceptions of medications especially about its necessity and concerns about its safety.

It was apparent from this study that treatment-related perceptions of participants were similar to those reported by other studies including those from Western cultures.^21,22^ Safety concerns about medications were identified as major barriers for medication adherence that led to refusal to initiate insulin out of concern of adverse effects or decreasing of doses and discontinuation of medications upon experience of adverse effects. These findings were similar to reports from other studies in Ethiopia as well as from other settings.^12,14,16,21,22,23,24^ On the other hand, illness perceptions about the impending complications and the chronicity of diabetes that were mediated with strong perceptions about the necessity of medications were observed to facilitate adherence to anti-diabetic medications. These findings were also similar to other studies that reported positive association between these factors and adherence to medications for long-term conditions including diabetes.^25,26,27^

With regard to illness perceptions especially those related to symptoms (e.g. hoping for a cure and treatment), it was evident that study participants hold explanatory models that were different from the biomedical model and those reported among Western cultures. These explanatory models seemed to be heavily influenced by participants’ cultural and religious backgrounds and appeared to be responsible, at least in part, for decisions and practices in managing diabetes.^28^ Among their illness perceptions which seem to affect adherence to recommended medication regimens were their perceptions about diabetes symptoms whose absence has led to delay in treatment or discontinuation of medications only to restart on them when symptoms reappear. Such observations are not common among Western patients diagnosed with diabetes although there are similar reports among immigrants in these countries and from Thailand. In these studies, participants expressed belief that diabetes was a short term condition with the oral anti-diabetic agents providing symptomatic relief but were unneeded once they ‘felt well’,^21,29,30,31^ only to restart on them when their illness was visible and possibly affecting their daily routines.^32^ Such reports are similar to reports from studies conducted among Ethiopian patients with diabetes which also reported ‘feeling of being well without treatment’ and ‘disappearance of symptoms’ as reasons for low adherence to anti-diabetic medications.^12,33^ This finding is also supported by other studies from the Ethiopian context where more emphasis is given to symptomatic, acute conditions and where hope for a cure is expressed.^34,35^

Hope for a cure especially in relation to religious healing was another factor that served as a barrier to anti-diabetic medication adherence among some study participants. In this case, the practice that involved going to holy water sites has led to the discontinuation of anti-diabetic medications for short or long durations among the followers of Orthodox Christianity. This may be related to the wide belief among the followers of Orthodox Christianity that supernatural forces (especially the devil) cause illnesses and that God provides healing for which holy water is a commonly used healing mechanism. Holy water is believed to provide cure when it is bathed in or drunk.^36^ Going to holy water sites to be baptised and healed has been one of the commonly cited reasons to discontinue treatment and being lost to follow up in favour of holy water is not uncommon according to studies conducted among patients with HIV.^37,38^ Such cases of discontinuation of chronic treatment in favour of alternative treatments are not unique to Ethiopia and are also reported from other settings in Africa. Studies conducted among patients with diabetes in Ghana and Tanzania had reported about hope for a cure that led to healer shopping that involved using faith healing (mainly prayers) and ethnomedicine which were used on a complementary basis but also as an alternative that led to discontinuation of the biomedical treatment.^39,40^ The alternative and wide use of holy water as religious healing practice, however, seems to be more common among the Orthodox Christians in Ethiopia.

In relation to religious healing, fasting while taking hypoglycaemic medications such as insulin and glibenclamide was observed to be a barrier to follow recommended medication schedules that could lead to problems in the long or short term. Fasting is actually a common routine among religious adherents in Ethiopia that is also considered by some to have a curative power.^37^ For the Orthodox Christians, 250 days of fasting are recommended of which 180 are obligatory for anyone above the age of 13. Fasting in general implies just one meal per day that is to be taken after 2:45 in the afternoon or in the evening and total avoidance of animal products such as meat, dairy products and eggs.^41^ In a similar manner, Muslims avoid foods or drinks from early dawn to after sunset during the month long Ramadan. Studies conducted among Ethiopian patients with HIV have reported how Orthodox Christians and Muslims either discontinued their antiretroviral treatment during the fasting seasons in their bid to fulfil religious duties but also to avoid stigma^37^ or modified their dosing schedules which in most cases involved missing one of their doses of anti-retroviral medication during fasting.^38^ This is among the first reports to report on the influence of fasting on anti-diabetes medication regimen among the followers of Orthodox Christianity although there exist literature that describe how Muslim patients would reduce their medication dose during the month of Ramadan.^21^

Conversely, religious and religious healing practices that included going to church, praying and even use of holy water at home were observed to provide some participants at the very least with psychological benefits which can positively influence the illness experience and adherence to medications albeit in an indirect manner. The psychological and mental benefits that these participants may have from the religious healing can minimise possible diabetes related distress that may be present in some of the participants that in turn may lead to lower adherence among other things^16,42,43^ as also practised by patients with HIV elsewhere in Ethiopia where the use of holy water alongside medications has been reported to lead to gains from a holistic treatment that may offer psychological, spiritual or mental health benefits.^37^ This seems in line with the concept of health in Ethiopia which is considered a holistic concept encompassing physical but also spiritual, social, mental and psychological aspects and that health is a ‘gift’ or ‘will’ of God and thus the importance of religion which many believe helps to keep them in good health.^36^ The use of religious practices among patients with diabetes is also reported from other settings such as among black people in the United States and participants from Iran where prayers, ‘turning things over to God’, reading Holy Scriptures and attending churches and mosques have been reported as coping strategies.^44,45^ The finding from Iran, where participants have engaged in ‘high levels’ of religious practices, has reported significant association between religious practices and self-care activities among the study participants.^45^ Religious coping mechanisms such as prayers and performing *solat* (obligatory prayers of Islam) which were described as ways of being close to God were also reported to be significantly correlated with and were mediators between illness perceptions and health-related quality of life positively in a study which involved patients with end-stage renal disease from Malaysia. This led the authors of this study to recommend that due attention be given to illness perceptions and positive religious coping mechanisms in intervention programmes to improve the quality of life of the patients.^46^

The findings of this study could be limited by the fact that only the perceptions of a group of patients, that is, those who were following treatment in public hospitals of urban centres were included and thus may not represent a more diverse group. Thus, the results may not be applicable to patients with diabetes who follow treatment in the health institutions outside the public arena as well as those who chose to avoid Western treatment. Nevertheless, this study included patients with diverse backgrounds in terms of socio-demographic, illness and treatment characteristics, which has enabled a rich and diverse view on their treatment experiences and perceptions about the facilitators and barriers to anti-diabetic medications.

### Practice implications

This study has practice implications especially about the need to provide appropriate patient education, be it in groups or individually, with regard to diabetes and recommended medications. This education should consider local and patient-specific contexts. This may include strengthening patients’ understanding of the chronicity of diabetes and stressing the necessity of their medications in relation to its benefits in reducing and delaying diabetes complications and improving outcomes. Patient education can also focus on the controllability of diabetes and about the meaning of symptoms, the relationship with blood glucose levels and the need to continue taking medications even if ‘symptoms’ disappear. This should be coupled with appropriate medication-related education that should emphasise their necessity while giving due attention to the possible adverse effects and how they can be prevented and mitigated. Healthcare providers should also closely monitor their patients with regard to their medication-taking experiences and make concerted effort to explore patients’ experiences of the medications including about any adverse effects with the objective of alleviating their concerns so as to support patients’ decisions to adhere to them.

Issues in relation to religious healing and practice require the recognition that some patients may have strong feelings about their religious practices and the need to adhere to them. Such issues require an open discussion about the pros and cons of religious healing and practices to be followed and the collaboration with religious healers and the religious leaders and teachings in order to support patients’ adherence to recommended medications while still practising religious healing and practices in a compatible manner. Attempts are reported of collaborative works in the areas of HIV/AIDS and psychiatric treatment with the Ethiopian Orthodox Church to encourage the use of both types of treatment citing compatibility, which have yielded encouraging results in terms of increased adherence to recommended medications.^37,47^ Similar attempts may also be beneficial for patients with diabetes as has also been hinted in the Malaysian study to improve patient outcomes.^46^ The issue with determining the benefits of religious healing and practice in relation to alleviating diabetes related distress and other issues such as fasting, however, are among areas that may need further research.

## Conclusion

A number of factors that could serve as barriers and facilitators to anti-diabetic medications adherence were identified. The major perceptions are about diabetes including its symptoms and curability, which seem to be different from that reported from Western culture, and concerns about the safety of recommended medications similar to those reported elsewhere. The findings are strongly suggestive of the need to institute strengthened education programmes for the patients on different topics such as diabetes and the anti-diabetic medications that are considerate of local and individual patient contexts. Further studies are recommended to explore the benefits of religious healing practices and the effects of fasting and other rituals among religious adherents.
